# Factors Influencing Health Care Professionals' Perceptions of Frequent Drug–Drug Interaction Alerts

**DOI:** 10.1055/s-0044-1782534

**Published:** 2024-03-31

**Authors:** Yasmine Biady, Teresa Lee, Lily Pham, Asad Patanwala, Simon Poon, Angus Ritchie, Rosemary Burke, Jonathan Penm

**Affiliations:** 1 School of Pharmacy, The University of Sydney Faculty of Medicine and Health, Sydney, New South Wales, Australia; 2 Department of Pharmacy, Royal Prince Alfred Hospital, Camperdown, New South Wales, Australia; 3 School of Computer Science, Faculty of Engineering, The University of Sydney, New South Wales, Australia; 4 Department of Nephrology, Concord Repatriation General Hospital, Sydney, New South Wales, Australia; 5 Department of Pharmacy, Sydney Local Health District, Sydney, New South Wales, Australia; 6 Department of Pharmacy, Prince of Wales Hospital, Randwick, Australia

**Keywords:** drug interactions, health care professionals, medication, alert systems

## Abstract

**Background** Drug–drug interactions (DDIs) remain a highly prevalent issue for patients in both community and hospital settings. Electronic medication management systems have implemented DDI alerts to mitigate DDI-related harm from occurring.

**Objectives** The primary aim of this study was to explore factors that influence health care professionals' (hospital doctors, hospital pharmacists, general practitioners, and community pharmacists) perceptions and action taken by them in response to DDI alerts.

**Methods** A qualitative study was conducted using semi-structured interviews between early January and late February 2021. The top 20 most frequently triggered DDI alerts previously identified were used as examples of alert prompts shown to participants.

**Results** A total of 20 participants were recruited. General practitioners ( *n* = 4) were most likely to consider DDI alerts to be clinically relevant and important, and hospital doctors ( *n* = 4) were most likely to consider these alerts not being clinically relevant nor important. Three main factors were identified to influence health care professionals' perceptions of DDI alerts, which included clinical relevance, visual presentation, and content of alerts.

**Conclusion** Health care professionals' perceptions of DDI alerts are influenced by multiple factors and considerations are required to create tailored alerts for users and their clinical contexts. Improvement in DDI alerts should be a priority to improve patient medication safety and health outcomes.

## Background and Significance

Drug–drug interactions (DDIs) remain a highly prevalent issue for patients in both community and hospital settings. 1 To address this, electronic medication management systems have implemented clinical decision support (e.g., DDI alerts) to mitigate DDI-related harm from occurring. 2 3 When active, these alerts generate interruptive messages for health care professionals when selecting a medication that could interact with another medication the patient is taking during the prescribing or dispensing process. They advise on the nature of the interaction, potential adverse outcomes due to the interaction, and often include a recommendation for monitoring, a change of dose, or advice to cease a medication. 4

The evidence regarding DDI alert acceptability and usability is mixed. Health care professionals typically prefer alerting systems over other DDI detection tools (e.g., look-up tools) 2 and DDI alert implementation is generally favored. 5 Specifically, tools that provide automatic recommendations and intervene at the time of decision-making where identified as effective. 6 However, users may experience alert fatigue and a high frequency of false-positive alerts, resulting in up to 90% of DDI alerts being overriden. 7 8 9 10 Low clinical relevance, 8 11 12 13 poor visual design, 14 15 and inconsistencies in drug knowledge databases 16 17 have been attributed to high rates of DDI alert overrides overall, in both international and Australian settings. A large proportion of the existing literature that explores user perceptions of DDI alerts comment on the system as a whole. 18 19 Although more recent studies have explored such perceptions in relation to specific DDI alerts, these perceptions of prescribers and dispensers have not been explored across community and hospital practice settings. 10

## Objectives

The primary aim of this study was to explore the factors that influence health care professionals' (hospital doctors, hospital pharmacists, general practitioners, and community pharmacists) perceptions and action taken by them in response to DDI alerts.

## Methods

### Interview Design

A multidisciplinary team with expertise in health informatics, pharmacy, and medicine conceived the study, developed the semi-structured interview guide, and interpreted the findings. The Standards for Reporting Qualitative Research were used as a guideline for this study ( Supplementary Appendix 1 , available in the online version).

The semi-structured interview guide included screen-captured images of 20 specific DDI alert interfaces to understand perceived clinical relevance of DDI alerts, any clinical considerations made by clinicians, as well as suggestions for DDI alert improvements. To elicit participants' realistic responses to and explore their experiences with common DDI alerts seen in practice, the top 20 most frequently triggered DDI alerts in an Australian teaching hospital, previously identified by Gatenby et al, 20 were used as examples of DDI alert prompts shown to participants ( Supplementary Appendix 2 , available in the online version). The screen-captured alerts were obtained from Cerner PowerChart which implements the Cerner Multum drug knowledge database. All 20 selected DDI alerts were identified in a hospital setting and classified as “major-contraindicated” alerts as defined by the Multum drug knowledge database. The Cerner PowerChart interface was selected due to its extensive use in hospital settings across Australia. Each investigator extensively reviewed the interview guide prior to its use.

Each participant was asked to observe each alert and answer the same four follow-up questions ( Supplementary Appendix 3 , available in the online version). Clinicians were asked to categorize the clinical relevance of DDI as either (1) clinically relevant and important, (2) clinically relevant, and important but of low priority, (3) not clinically relevant or important, or (4) further investigation and information required. They also categorized their actions based on the DDI alert as either (1) follow alert advice, (2) override, or (3) further investigation and information required.

### Participant Recruitment

Hospital clinicians were sent an invitation email to participate in January 2021 from the Chief Medical Information Officer and Director of Pharmacy of a tertiary teaching hospital. For primary care clinicians, emails were sent to 51 medical practices and 39 community pharmacies near the tertiary teaching hospital. Email addresses were obtained by from publicly available sources. To be eligible for study inclusion, clinicians interviewed must use DDI alerts systems and be a medication prescriber or dispenser.

One reminder email with the participant information sheet and consent form were sent to each acquired email address after 2 weeks. All interested candidates who responded to follow-up queries were contacted via email by the research team and supplied with a participant information sheet. Written consent was obtained prior to interview. Passive snowball sampling was used to facilitate recruitment. Participants were offered a $50 gift card for their time.

### Data Collection and Analysis

Interviews were conducted between early January and late February 2021. Each interview was conducted over Zoom and lasted approximately 60 minutes. One investigator (Y.B.) conducted each interview and took observation notes throughout each meeting. Interviews were conducted until data saturation was reached. Data saturation was determined when additional interviews no longer produced new themes or codes. 21

Interviews were audio-recorded and transcribed verbatim by a member of the research team (Y.B.). All transcripts were de-identified of sensitive information relating to each participant, such as names and places of employment. Participants were given the option to review their interview transcript prior to its use in the study. Transcripts underwent inductive thematic analysis in an iterative manner, allowing themes and subthemes to be identified. 22 All transcripts were first inductively coded by two independent researchers (Y.B. and T.L.) using NVivo 11 (QSR International Pty Ltd., released 2015, Version 1.0.1.1). Both researchers, who are pharmacy honors students with limited clinical experience, discussed differences in coding until consensus was reached. 23 Codes were further grouped into themes and subthemes by the research team.

Where clinicians were asked to categorize the clinical relevance of a DDI and their action in response to such alert, these data were analyzed quantitatively.

## Results

A total of 20 participants were recruited for the research study which included community pharmacists ( *n* = 6), hospital pharmacists ( *n* = 6), general practitioners ( *n* = 4), and hospital doctors ( *n* = 4). Roles of the participating hospital doctors included a geriatric advanced trainee, a renal registrar, a junior medical officer, and an anesthetics co-director.

Overall, general practitioners were most likely to consider a DDI alert to be clinically relevant and important to a patient, hospital pharmacists were most likely to consider DDI alerts to be clinically relevant but of low priority, hospital doctors were most likely to consider DDI alerts as not being clinically relevant nor important, and community pharmacists often required further investigation or information to inform their decision.

General practitioners were most likely to follow alert advice, hospital pharmacists were most likely to override alerts, and community pharmacists were most likely to seek further information before action.

Three main themes of clinical relevance, visual presentation, and content (see Table 1 ) were identified as factors influencing health care professionals' perceptions of DDI alerts.

**Table 1:** List of themes influencing health care professionals' perceptions of DDI alerts

Factors influencing health care professionals' perceptions of DDI alerts		
*Theme 1: Clinical relevance of alerts*		
	Subthemes	Codes
	1.1 Clinician factors	Customization to user
		Experience versus theoretical risk
	1.2 Patient factors	Prior use of combination
		Current health status
	1.3 Drug factors	Strength of dose
		Frequency of administration
		Route of administration
		Nature of the drug
		QTc prolongations
	1.4 Clinical setting	Community versus hospital
*Theme 2: Visual presentation of alerts*		
	Subthemes	Codes
	2.1 Text related	Amount of text
		Appearance of text
		Clarity and simplicity of text
		Organization of text
	2.2 Interface/display	Ability to draw attention
		Use of space
		Number of alerts
*Theme 3: Content of alerts*		
	Subthemes	Codes
	3.1 Quality of information	Specificity and accuracy
	3.2 Types of information	Integrate patient data
		Monitoring parameters
		Risk stratification information
		Alternative order suggestions

### Clinical Relevance of Alerts

The subthemes “clinician factors,” “patient factors,” “drug factors,” and “clinical setting” were derived from the theme, “Clinical relevance of DDI alerts.”

#### Clinician Factors

DDI alerts varied in clinical relevance depending on the health care professionals' area of expertise, level of seniority, and previous experiences.

*Customization to the user* : tailoring alerts to the individual use was mentioned by various health care professionals.

*“If you're in anaesthetics, you're always prescribing droperidol, prochlorperazine and ondansetron… but it's rarely prescribed by other people, so other people might need to consider it because it's a different clinical context.”* (HD4)

Participants mentioned the stark difference between how useful a junior doctor would find DDI alerts compared with a senior doctor, with one general practitioner stating that, *“It's good to have drug–drug alerts, particularly having been a junior doctor. You just chart things because you're told to chart it”* (GP1). Some participants mentioned that having the ability to silence alerts after receiving the same alert multiple times would be helpful:

*“You should be able to say, look, you've shown me this five times, I don't want to see it in the future.”* (HD4)

*Experience versus theoretical risk* : majority of participants mentioned a disconnect between the risk presented by DDI alerts and what they see firsthand in clinical practice. *“I've seen this multiple times… wouldn't even flag it with anyone.”* (HP4).

“ *A lot of interactions are theoretical interactions and if the patient has been on them together for a while and the doctor has been closely monitoring the risk … it may be considered okay and safe in that person.”* (CP4)

#### Patient Factors

Generally, DDI alerts were found to be less clinically relevant if patients have previously used a certain drug combination, or if the patient was in a stable condition.

*Prior use of the combination* : health care professionals felt more comfortable overlooking a DDI if the patient had previously used the drug combination without issue:

*“You might have patients that might have a contraindication [with their medicines], but they've been stable on the medication for a long period of time. So, taking time to look at the patient's history is quite valuable.”* (CP5)

Doctors stated that they felt more comfortable prescribing a drug combination if it had been previously initiated by another doctor, *“I would use it if it's already prescribed by someone else or they've already been on it, and then consider a repeat ECG… [but] I wouldn't start it if that alert came up”* (GP3).

*Current health status* : all health care professionals spoke about the importance of taking the patient's current health status into account when analyzing the relevance of a DDI alert, *“you consider the context of patient age, co-morbidities, other medications… so [you] go back and reassess the patient”* (GP1).

*“If my patient was over 60 years old and was a cancer patient on very high doses of domperidone and was taking a CYP3A4 inhibitor and had some electrolyte disturbances, I'd be far less willing to chart this medication.”* (HD3)

#### Drug Factors

*Strength of dose* : all participants considered DDI alerts to be more clinically relevant if patients were taking high doses.

*“If someone was on high doses, then yes, it becomes clinically relevant.”* (HP2)

*Frequency of administration* : clinicians believed DDI alerts to be more useful if the medication combination was taken on a regular basis and over a long period of time.

*“Someone using buprenorphine PRN, I wouldn't be as worried if they were using it as a regular thing.”* (HD1)

*Route of administration* : DDI alerts for medications administered by parenteral routes, such as intravenous formulations and depot injections, prompted health care professionals to be more cautious.

*“The amount of drug you absorb when you inhale is so small that it's just theoretical... you might worry if you were giving an intravenous salbutamol infusion.”* (HD4)

*Nature of the drug* : participants mentioned that certain medications, such as haloperidol, were considered to be inherently “ *riskier* ,” with some noting certain drugs as *“dirty drugs”* (HP5) and therefore taking closer note of a DDI alert.

*QTc prolongation interactions* : many participants recognize the importance of QT-prolonging drugs due to the potentially fatal outcomes, however, rarely is it an issue for a vast majority of patients.

*“QT interval, it's one of those things where I feel that it's very relevant … but usually it's not an issue for the patient.”* (HP2)

For doctors prescribing QT-prolonging drugs on a regular basis, DDI alerts were a major source of frustration.

*“The standard regime of drugs to prevent nausea and vomiting include medications, which in theory, prolong the QT interval… almost on a daily basis, we get these interactions that come up and we just click past … it's just an annoyance.”* (HD4)

#### Clinical Setting

Overall, health care professionals practicing in the community considered the DDI alerts to be of higher clinical relevance than those practicing in hospital settings.

*Community versus hospital* : overall, clinicians practicing in the community believed that DDI alerts were relevant to reduce patient harm but found it difficult to determine the clinical significance. Many considered QT prolongation to be out of their scope of practice and capability.

*“I can't ever recall hearing a patient come in with saying they've had an issue with their QT interval in a community pharmacy.”* (CP4)

*“I wouldn't prescribe it in the GP setting. I wouldn't be starting a new antipsychotic in a patient.”* (GP1)

Hospital prescribers often did not consider DDI alerts to be particularly useful due to the familiarity of prescribing certain drug combinations.

*“This would be one of those ones where, where we would just click straight past. Prochlorperazine is part of our standard post-operative nausea and vomiting protocol.”* (HD4)

The availability of prompt monitoring services in hospital settings influenced the attitude hospital pharmacists displayed toward DDI alerts.

*“Patients in the hospital setting, because it's an acute setting, are being monitored closely so, I will take it [DDI alerts] with a grain of salt.”* (HP4)

### Visual Presentation of Alerts

The subthemes, “text related” and “interface and display” were derived from the theme “visual presentation of alerts.”

### Text Related

Many visual aspects of the text embodied within a DDI alert strongly influenced health care professionals' desire of whether to read or ignore the alert.

*Amount of text* : health care professionals often considered alerts to be hard to read and found it difficult to extract salient points.

*“There's far too much text and it's not formatted in a way that's easy to skim.”* (HD3)

*Appearance of text* : font size was frequently discussed by participants, often mentioning that the text was too small.

*“I think the style of writing, the font, the sizing, and just where it's placed, I don't think it particularly triggers anything.”* (HP1)

Community pharmacists and general practitioners held negative perceptions toward tall-man lettering.

*“Some of the letters are in capital letters and some are in lowercase… I'm not sure what they're trying to highlight by doing that.”* (GP1)

*“The capital CHLORPER, and small azine annoys me… I'd rather it was consistent.”* (CP1)

*Clarity and simplicity of text* : many health care professionals found that the DDI alert did not clearly explain the mechanism and severity of the DDI, with some participants disagreeing with the rationale given by the DDI alert for certain DDIs.

*“I just think like it says, ‘This should not be used longer than 12 weeks except in rare cases when therapeutic benefits…’, I just feel like that's confusing advice to give.”* (CP3)

*Organization of text* : health care professionals preferred DDI alerts to begin with a summary of important information required to facilitate quick decision-making.

*“If they're more succinct and more concise then you can possibly handle the situation quicker.”* (CP3)

*“Even if you're going to provide the same information in dot points, it would still make my life easier.”* (HP4)

#### Interface and Display

Aspects of the DDI alert interface, including the visual display and the frequency of alerting, were also identified.

*Ability to draw attention* : the use of color to indicate severity, increasing text size and bolding text to emphasize important information of the medication order were suggestions to improve DDI alerts.

*“It's worthwhile for things that are definitely contraindicated [to have] big red, bold letters so it stops you in your tracks straight away.”* (GP1)

*Use of space* : participants frequently commented on the suboptimal utilization of space within the DDI alert.

*“You have a lot of spare space on the right-hand side of the screen. You can maybe fit in some history there.”* (CP1)

*Number of alerts* : participants agreed that the frequency and amount of DDI alerts triggered in respective software systems negatively influenced their perceptions toward alerts, often citing them as “ *frustrating* ” and “ *annoying* .”

*“The more that you see it the more you think maybe that it's OK because if all of them are popping up ‘Major’ maybe it reduces the…I guess, how important it might be.”* (HP3)

### Informational Content of Alerts

The subthemes “quality of information” and “type of information” were derived from the theme “informational content of alerts.”

#### Quality of Information

*Specificity and accuracy* : health care professionals commented that the information included in DDI alerts was too broad and generalized to facilitate effective decision-making. Frequent complaints were that the same information was repeated for different DDI alerts, therefore appearing as blanket statements for classes of medications with a lack of specificity to the implicated drugs in the DDI. Participants identified that they were more likely to ignore alert content and advice as a result.

*“The management is very generic… “this drug is considered contraindicated with every drug that can prolong QT interval” is far too broad for me. I want more detailed information on those two agents… when you get statements that are theoretical and are based on classes of medications as opposed to the specific agents, I'm personally not likely to pay as much attention to it.”* (CP4)

#### Types of Information

Only certain types of information were found to be useful in facilitating decision-making in response to a DDI alert.

*Integrate patient data* : there was a resounding consensus for DDI alerts to include patient-specific information within the alert screen, such as their medication history, comorbidities, and pathology results.

*“We only know the two drugs here. We don't know the whole entire patient's history in this particular case… you're only seeing bits of the story.”* (CP3)

*Monitoring parameters* : participants expressed a desire for DDI alerts to list specific recommendations for parameters to be monitored.

*“I think there should also be something about monitoring, like serial ECG monitoring for QT prolongation… electrolyte monitoring for potassium and magnesium.”* (HD3)

*Risk stratification information* : participants wanted DDI alerts to explain clear differentiations between severity levels and include information pertaining to the likelihood of a patient experiencing harm from the interaction.

*“The problem with just saying that it's a major interaction is, it's major for who? For how many people? Is it major for one in a hundred people, one in a thousand? One in ten thousand? Give some kind of guidance on the likelihood.”* (CP4)

Most participants commented that they would find DDI alerts more useful and effective if recommendations for alternative agents were provided.

*“It's always good if you give me an example of a non-contraindicated drug. Cause the most frustrating thing would be, if I put a Stemetil or Maxolon and then it comes up again.”* (GP3)

## Discussion

This study explores factors influencing prescribers' and dispensers' perceptions of DDI alerts in Australian community and hospital practice settings. Specifically, it has outlined how the design of future clinical decision supports requires increased user adaptability to meet user needs by identifying that health care professionals' perceptions of DDI alerts are influenced by their clinical relevance, visual display and presentation, as well as informational content. Clinicians in this study recognized that major improvements are required in all three aspects to improve the user experience and increase alert effectiveness. Other studies have echoed this sentiment, citing that DDI alerts are better received, less likely to be overridden, and more effective if they contained more information tailored to the patient, 24 25 user, 26 27 28 and the clinical context in which DDI alerts are used. 14 An evaluation of DDI alerts, including of the Cerner PowerChart interface in an Australian context, has highlighted the need for further consideration of human factors principles of good warning design, 29 further supporting health care professionals' perceptions of factors influencing their actions following a DDI alert. While tools including the Instrument-for-Evaluating-Human-Factors-Principles-in-Medication-Related-Decision-Support-Alerts (I-MeDeSA) have been effective in identifying improvements for effective DDI alert system design according to user preferences, 4 14 30 31 such tools are primarily developed for the U.S. market and may require some modification for increased usability and applicability to the Australian context. 29 Further research in this space may allow for the development of DDI alert systems that consider the clinical relevance, visual display, and presentation, as well as informational content of DDI alerts in Australian community and hospital practice settings.

Variability between hospital and primary care clinicians' views of DDI alerts differed with community-based health care professionals more likely to consider the DDI alerts to be clinically relevant and important when compared with their hospital-based contemporaries. These findings have been reflected in other studies where clinicians in primary care considered some DDI alerts to not be meaningful, potentially due to the lack of specificity in the DDI alert systems to their practice setting. 32 33 Hence, clinicians practicing in community may exercise more caution in their decision-making due to their unfamiliarity with drug combinations commonly managed in hospital settings. Furthermore, the reduced availability to timely patient monitoring in community settings may further contribute to this level of caution. Although hospital clinicians may be more comfortable in overriding certain DDI alerts 16 potentially due to their perceptions of common DDIs being of low priority, or not clinically relevant nor important, literature also suggests that these clinicians may frequently prescribe medicines despite being unfamiliar with their associated DDIs. 34 Rather, health care professionals were guided by their firsthand experience in clinical practice with regard to clinical decision-making, potentially contributing to wide variations in decision support uptake and acceptability. 35 36 User-tailored alerts, including changes in timing to prompt during medication charting rather than on order submission, as well as alerts filtering for more severe potential interactions were suggested to prevent alert fatigue and promote appropriate clinical evaluation. 34 37 Considerations for tailored usability may address the differences in DDI clinical decision-making behaviors across community and hospital settings, as well as between doctors and pharmacists. 33

## Limitations

Although the smaller sample size ( *n* = 20) may have presented as a limitation to this study, interviews were conducted until data saturation was reached while also capturing a diverse range of community and hospital health care professionals. As a result, the findings of this study are generalizable to the clinician experiences with these DDI alerts. Participants in this study were also shown a specific selection of DDI alerts based on prior research conducted at the same site. Although DDI alerts selected for this study were chosen because they are the most commonly encountered by clinicians, over 80% of the included DDI alerts' adverse effects are specific to QTc prolongation and extrapyramidal symptoms. 20 Consequently, the results of this study may be selective to the specific drug combinations shown and may not be generalizable to the wider perceptions of DDI alerts overall. Furthermore, recruitment of participants for this study occurred within one local health district of a metropolitan city, which may not be indicative of the perceptions of health care professionals practicing in other contexts, such as in rural settings or with patient populations of different demographic characteristics. Further research that considers these differences in clinical practice, a range of DDI alerts, and the implications of the DDI alert effectiveness on patient outcomes should be undertaken.

## Conclusion

Health care professionals' perceptions of DDI alerts are influenced by three major factors: the clinical relevance of alerts, their visual display and presentation, and their informational content. Major reworking is required in all facets to create tailored alerts for users, as user experience and familiarity with drug combinations are seen to be a major contributing factor influencing alert acceptability amongst clinicians. Improvements in DDI alerts should be a priority to optimize alert effectiveness, facilitate clinician decision-making, and improve patient medication safety and health outcomes.

## Clinical Relevance Statement

This study identified factors that might affect health care professionals' perceptions of DDI alerts and therefore highlights the importance to creating tailored alerts.
