# Supplementary material for: Factors Influencing Health Care Professionals' Perceptions of Frequent Drug–Drug Interaction Alerts
Source: ACI open. 2024 Mar 31;8(1):e25–32. doi: 10.1055/s-0044-1782534 (PMC13404517; doi:10.1055/s-0044-1782534)
Supplement: Supplementary file 1 — Supplementary Material [file 10-1055-s-0044-1782534-s202306ra0010.pdf]

Supplementary Appendix 1 Standards for Reporting Qualitative Research (SRQR)[a]

Title and abstract		Page
	Title: concise description of the nature and topic of the study Identifying the study as qualitative or indicating the approach (e.g., ethnography, grounded theory) or data collection methods (e.g., interview, focus group) is recommended	Page 1
	Abstract: summary of key elements of the study using the abstract format of the intended publication; typically includes background, purpose, methods, results, and conclusions	Page 1
Introduction		
	Problem formulation: description and significance of the problem/phenomenon studied; review of relevant theory and empirical work; problem statement	Page 1–2
	Purpose or research question: purpose of the study and specific objectives or questions	Page 2
Methods		
	Qualitative approach and research paradigm: qualitative approach (e.g., ethnography, grounded theory, case study, phenomenology, narrative research) and guiding theory if appropriate; identifying the research paradigm (e.g., postpositivist, constructivist/ interpretivist) is also recommended; rationale[b]	Pages 2
	Researcher characteristics and reflexivity: researchers' characteristics that may influence the research, including personal attributes, qualifications/experience, relationship with participants, assumptions, and/or presuppositions; potential or actual interaction between researchers' characteristics and the research questions, approach, methods, results, and/or transferability	Page 2
	Context: setting/site and salient contextual factors; rationale[b]	Page 2
	Sampling strategy: how and why research participants, documents, or events were selected; criteria for deciding when no further sampling was necessary (e.g., sampling saturation); rationale[b]	Page 2
	Ethical issues pertaining to human subjects: documentation of approval by an appropriate ethics review board and participant consent, or explanation for lack thereof; other confidentiality and data security issues	Page 7
	Data collection methods: types of data collected; details of data collection procedures including (as appropriate) start and stop dates of data collection and analysis, iterative process, triangulation of sources/methods, and modification of procedures in response to evolving study findings; rationale[b]	Pages 2
	Data collection instruments and technologies: description of instruments (e.g., interview guides, questionnaires) and devices (e.g., audio recorders) used for data collection; if/ how the instrument(s) changed over the course of the study	Pages 2
	Units of study: number and relevant characteristics of participants, documents, or events included in the study; level of participation (could be reported in results)	Page 3
	Data processing: methods for processing data prior to and during analysis, including transcription, data entry, data management and security, verification of data integrity, data coding, and anonymization/de-identification of excerpts	Pages 2
	Data analysis: process by which inferences, themes, etc., were identified and developed, including the researchers involved in data analysis; usually references a specific paradigm or approach; rationale[b]	Pages 2
	Techniques to enhance trustworthiness: techniques to enhance trustworthiness and credibility of data analysis (e.g., member checking, audit trail, triangulation); rationale[b]	Pages 2
Results/findings		
	Synthesis and interpretation: main findings (e.g., interpretations, inferences, and themes); might include development of a theory or model, or integration with prior research or theory	Pages 3–6
	Links to empirical data: evidence (e.g., quotes, field notes, text excerpts, photographs) to substantiate analytic findings	Pages 3–6
Discussion		
	Integration with prior work, implications, transferability, and contribution(s) to the field: short summary of main findings; explanation of how findings and conclusions connect to, support, elaborate on, or challenge conclusions of earlier scholarship; discussion of scope	Pages 6

(*Continued*)

 © 2024. The Author(s).

Supplementary Appendix 1 (*Continued*)

	of application/generalizability; identification of unique contribution(s) to scholarship in a discipline or field	
	Limitations: trustworthiness and limitations of findings	Page 6–7
Other		
	Conflicts of interest: potential sources of influence or perceived influence on study conduct and conclusions; how these were managed	Page 7
	Funding: sources of funding and other support; role of funders in data collection, interpretation, and reporting	Page 7

[a]The authors created the SRQR by searching the literature to identify guidelines, reporting standards, and critical appraisal criteria for qualitative research; reviewing the reference lists of retrieved sources; and contacting experts to gain feedback. The SRQR aims to improve the transparency of all aspects of qualitative research by providing clear standards for reporting qualitative research.

[b]The rationale should briefly discuss the justification for choosing that theory, approach, method, or technique rather than other options available, the assumptions and limitations implicit in those choices, and how those choices influence study conclusions and transferability. As appropriate, the rationale for several items might be discussed together.

Supplementary Appendix 2 The top 20 DDI alerts as identified by Gatenby et al[20]

1. Metoclopramide AND prochlorperazine
2. Metoclopramide AND quetiapine
3. Potassium chloride AND spironolactone
4. Quetiapine AND ziprasidone
5. Amidotrizoate AND metformin
6. Buprenorphine AND droperidol
7. Metoclopramide AND olanzapine
8. Amiodarone AND ondansetron
9. Domperidone AND ondansetron
10. Droperidol AND tacrolimus
11. Droperidol AND escitalopram
12. Haloperidol AND quetiapine
13. Escitalopram AND prochlorperazine
14. Ziprasidone AND zuclopenthixol
15. Escitalopram AND quetiapine
16. Buprenorphine AND haloperidol
17. Citalopram AND prochlorperazine
18. Droperidol AND quetiapine
19. Droperidol AND mirtazapine
20. Domperidone AND salbutamol

 © 2024. The Author(s).

Supplementary Appendix 3 Interview Guide

I. General Information and Experience

1. Can you tell me about your role and how long you have been working for?
2. What has your experience been with drug–drug interaction alerts?

II. Drug Interaction Alerts

I am now going to present you with 20 drug–drug interaction alerts if time permits.

- *For hospital doctors and pharmacists → Ask all scenarios.*
- *For GP and community pharmacist → Do not ask about scenario 5, 6, 10, 11, 14, 18, 19.*

Scenario 1: <show alert for metoclopramide AND prochlorperazine (EPS or NMS)>

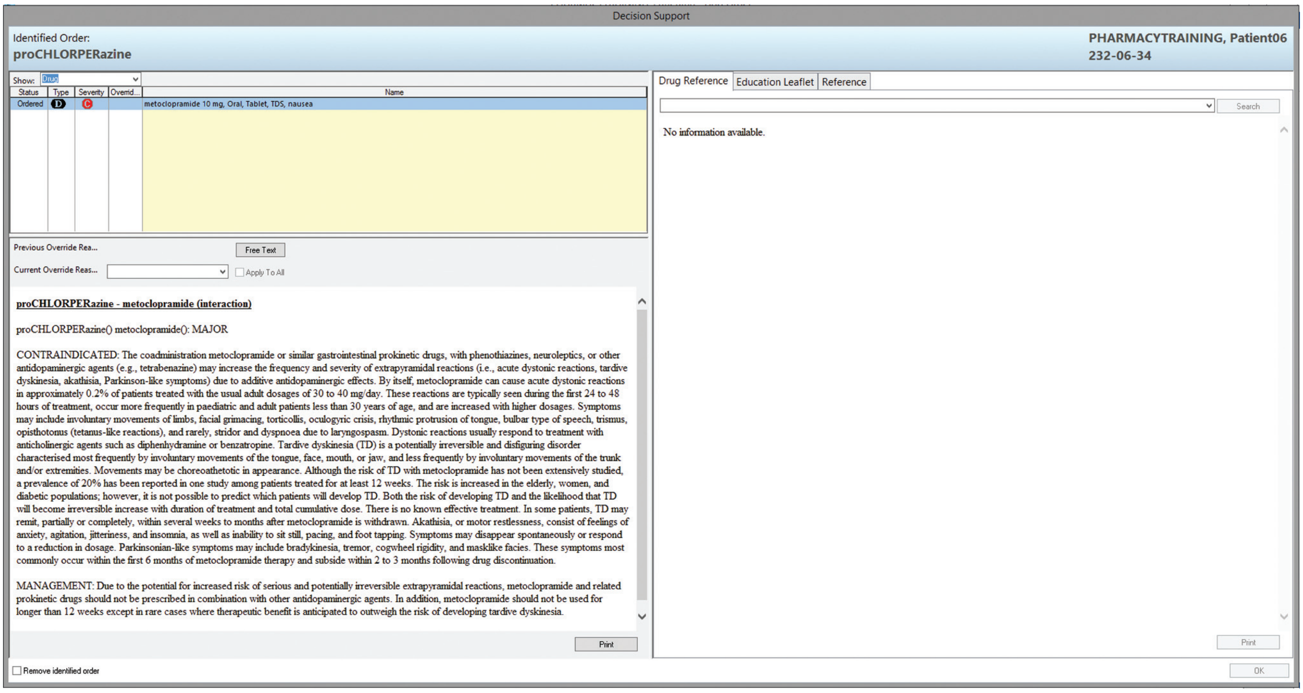

Fig 1

3a. What are your thoughts about the clinical relevance of this drug–drug interaction alert?
3b. What would you do if you saw this drug–drug interaction alert for one of your patients?
3c. Does anything frustrate you about this drug–drug interaction alert?
3d. How would you improve this drug–drug interaction alert?

Scenario 2: <show alert for metoclopramide AND quetiapine (EPS or NMS)>

 © 2024. The Author(s).

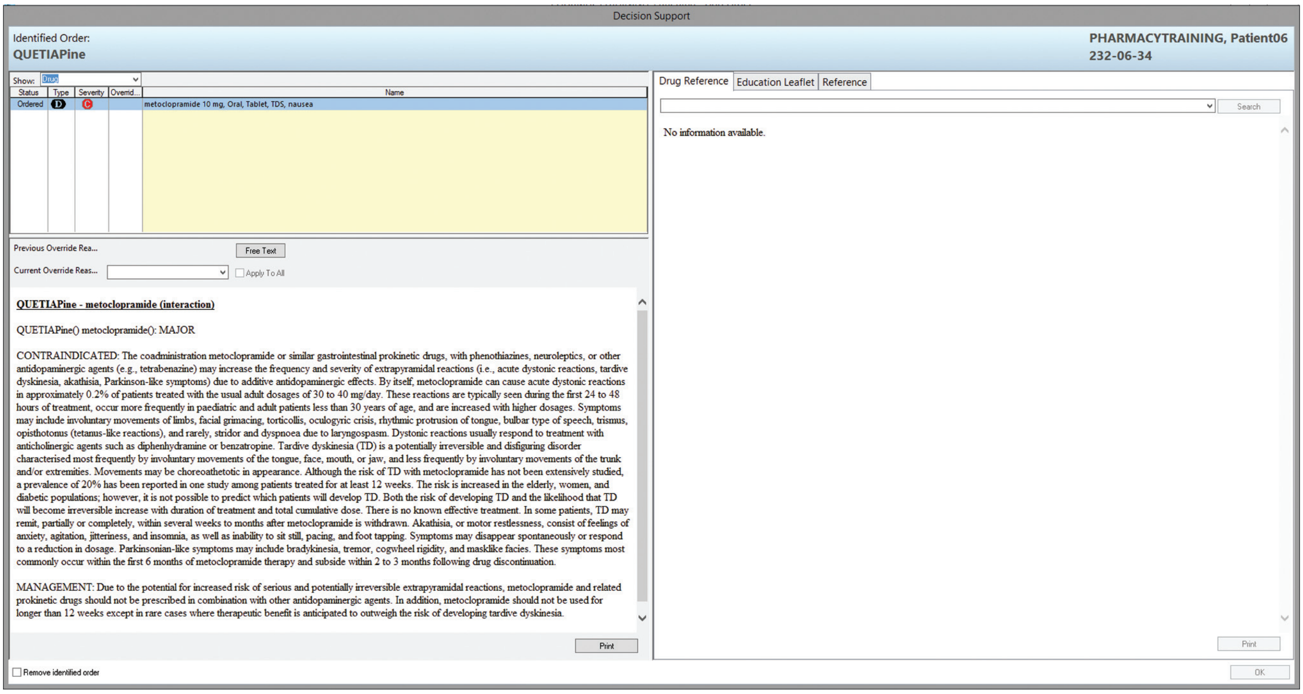

Fig 2

4a. What are your thoughts about the clinical relevance of this drug–drug interaction alert?
4b. What would you do if you saw this drug–drug interaction alert for one of your patients?
4c. Does anything frustrate you about this drug–drug interaction alert?
4d. How would you improve this drug–drug interaction alert?

Scenario 3: <show alert for potassium chloride AND spironolactone (Hyperkalemia)>

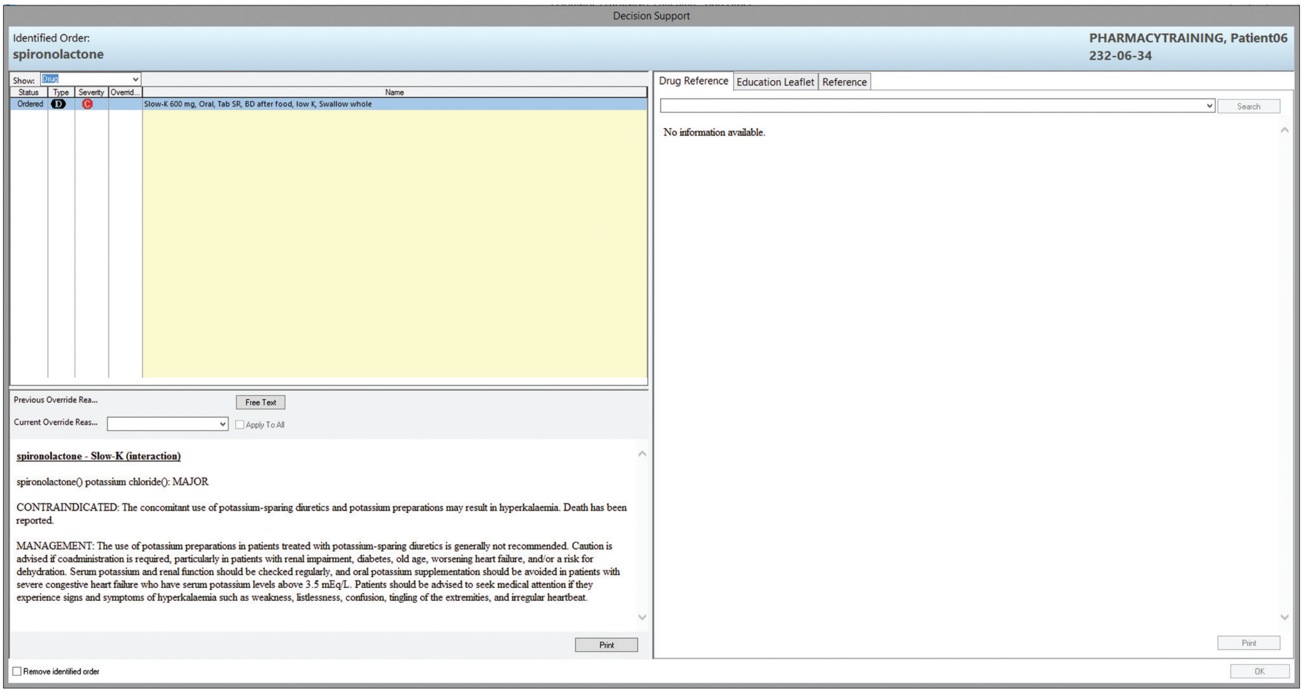

Fig 3

 © 2024. The Author(s).

5a. What are your thoughts about the clinical relevance of this drug–drug interaction alert?
5b. What would you do if you saw this drug–drug interaction alert for one of your patients?
5c. Does anything frustrate you about this drug–drug interaction alert?
5d. How would you improve this drug–drug interaction alert?

Scenario 4: <show alert for quetiapine AND ziprasidone (QTc and TdP)>

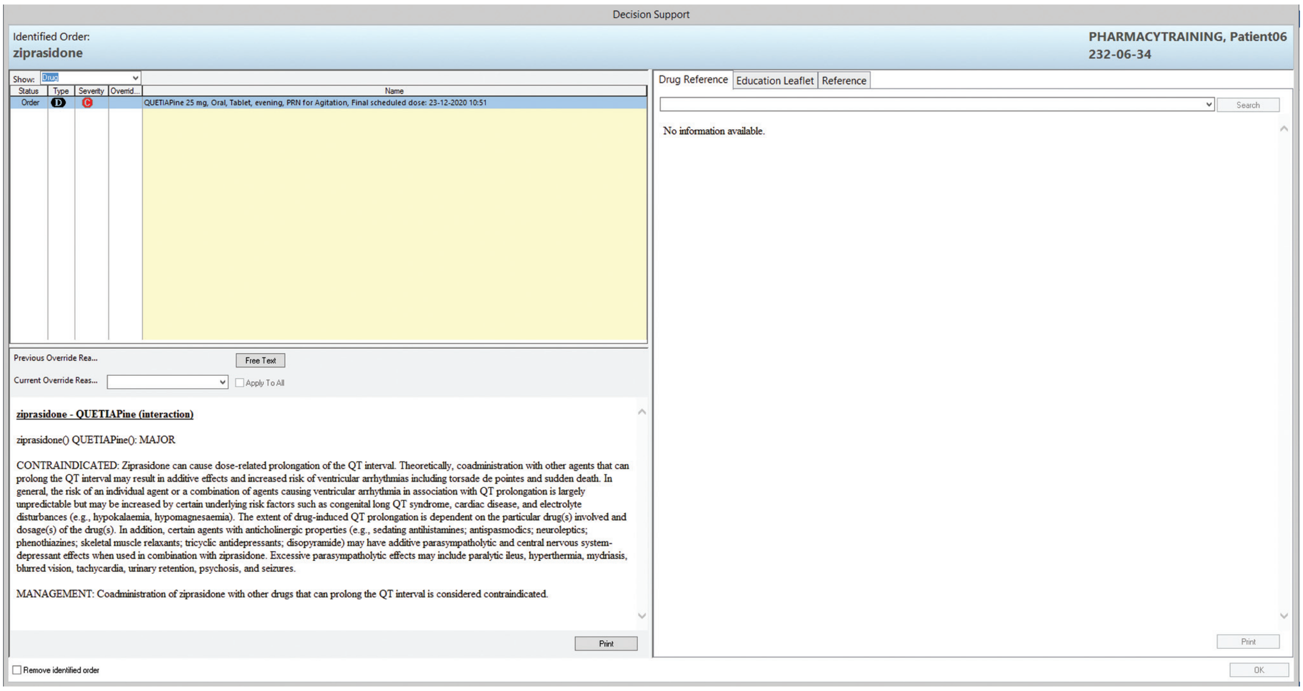

Fig 4

6a. What are your thoughts about the clinical relevance of this drug–drug interaction alert?
6b. What would you do if you saw this drug–drug interaction alert for one of your patients?
6c. Does anything frustrate you about this drug–drug interaction alert?
6d. How would you improve this drug–drug interaction alert?

 © 2024. The Author(s).

Scenario 5: <show alert for amidotrizoate AND metformin (Lactic Acidosis)>

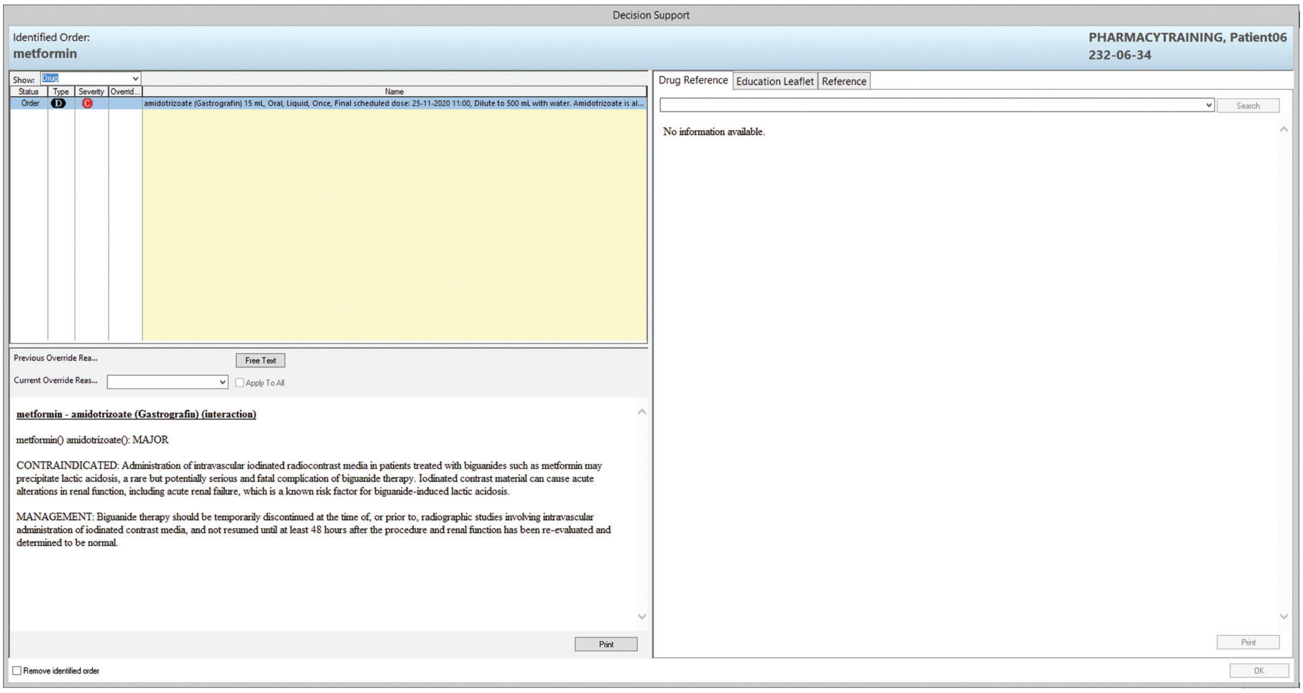

Fig 5

7a. What are your thoughts about the clinical relevance of this drug–drug interaction alert?
7b. What would you do if you saw this drug–drug interaction alert for one of your patients?
7c. Does anything frustrate you about this drug–drug interaction alert?
7d. How would you improve this drug–drug interaction alert?

Scenario 6: <show alert for buprenorphine AND droperidol (QTc and TdP, CNS Depression)>

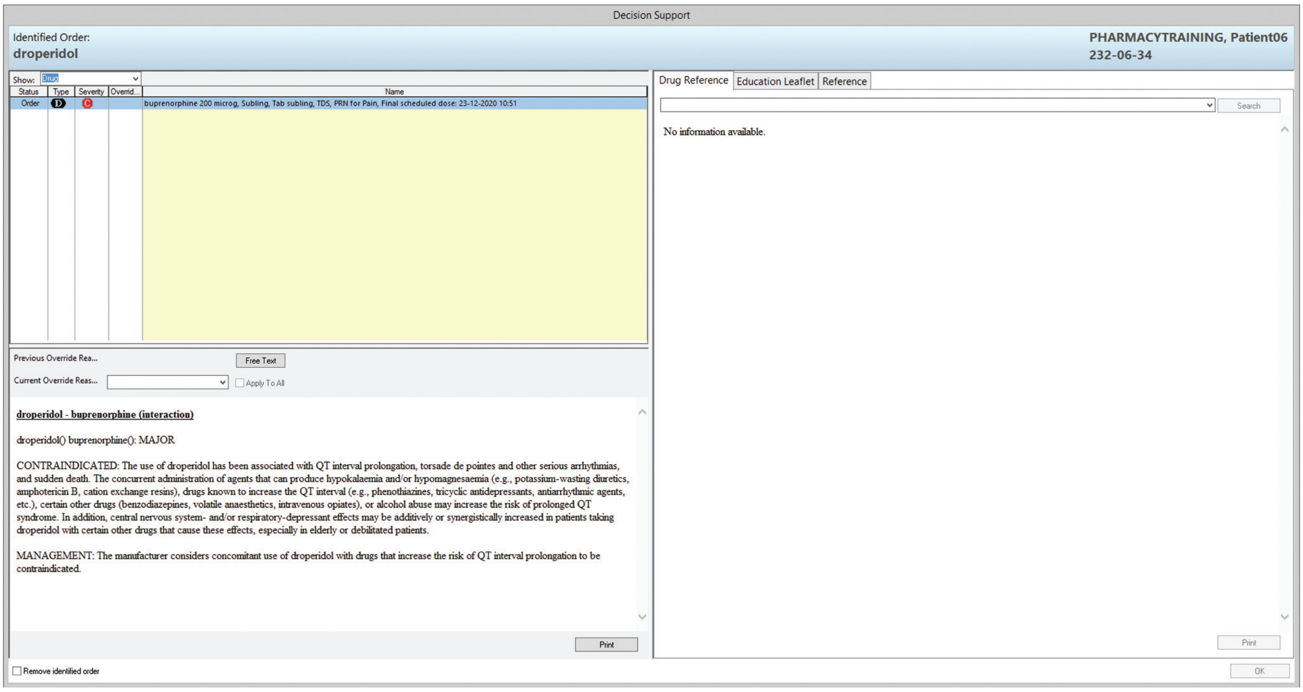

Fig 6

 © 2024. The Author(s).

8a. What are your thoughts about the clinical relevance of this drug–drug interaction alert?
8b. What would you do if you saw this drug–drug interaction alert for one of your patients?
8c. Does anything frustrate you about this drug–drug interaction alert?
8d. How would you improve this drug–drug interaction alert?

Scenario 7: <show alert for metoclopramide AND olanzapine (EPS or NMS)>

9a. What are your thoughts about the clinical relevance of this drug–drug interaction alert?
9b. What would you do if you saw this drug–drug interaction alert for one of your patients?
9c. Does anything frustrate you about this drug–drug interaction alert?
9d. How would you improve this drug–drug interaction alert?

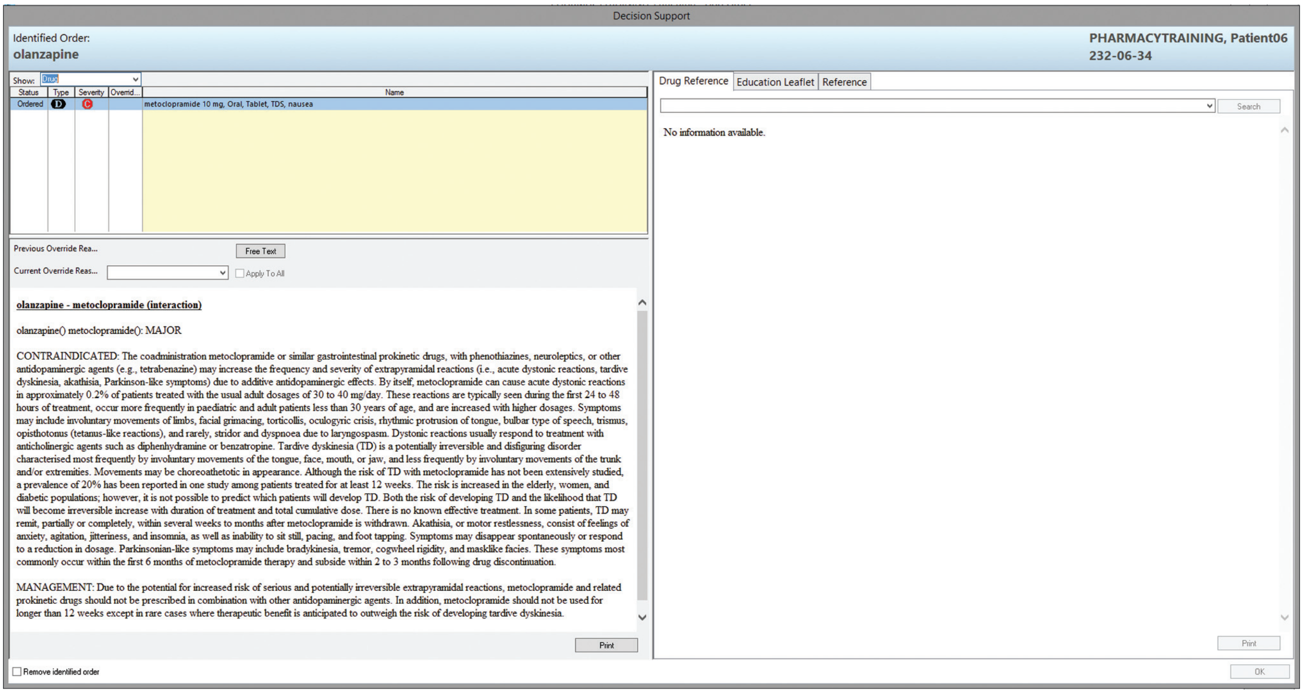

Fig 7

 © 2024. The Author(s).

Scenario 8: <show alert for amiodarone AND ondansetron (QTc and TdP)>

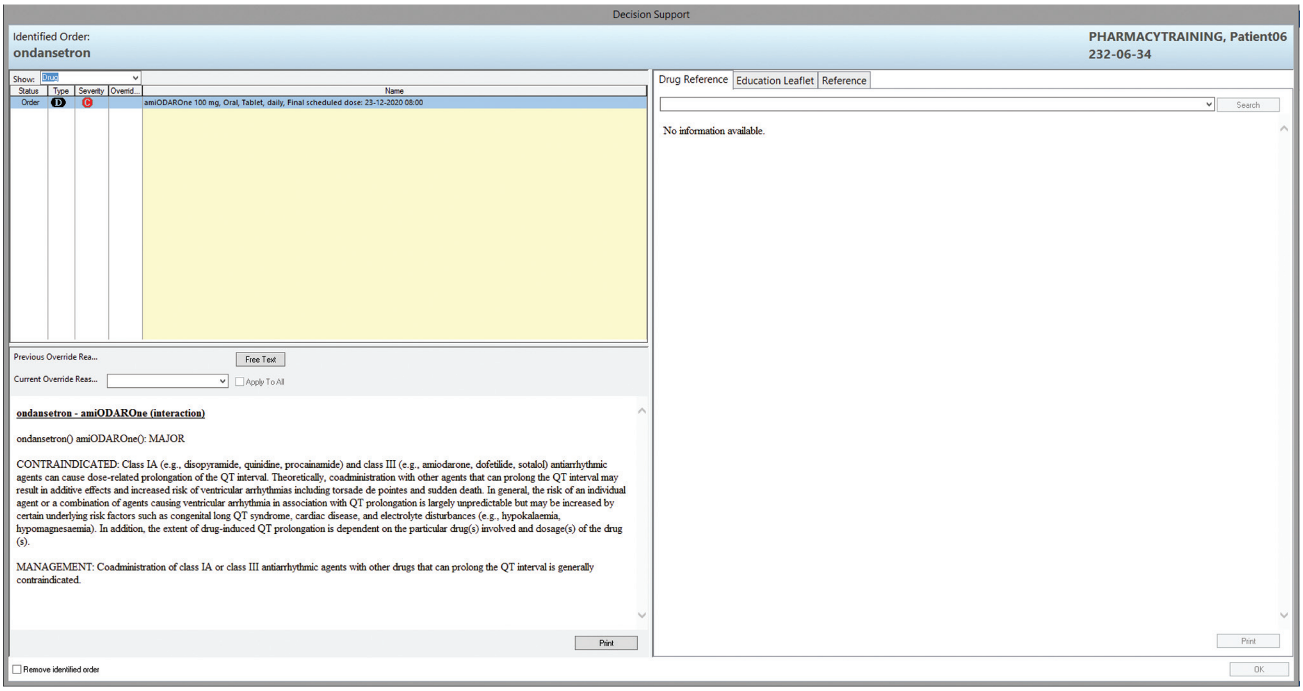

Fig 8

10a. What are your thoughts about the clinical relevance of this drug–drug interaction alert?
10b. What would you do if you saw this drug–drug interaction alert for one of your patients?
10c. Does anything frustrate you about this drug–drug interaction alert?
10d. How would you improve this drug–drug interaction alert?
IF TIME ALLOWS – Continue remaining scenarios as long as the participant is comfortable or 50 minutes has elapsed.

Scenario 9: <show alert for domperidone AND ondansetron (QTc and TdP)>

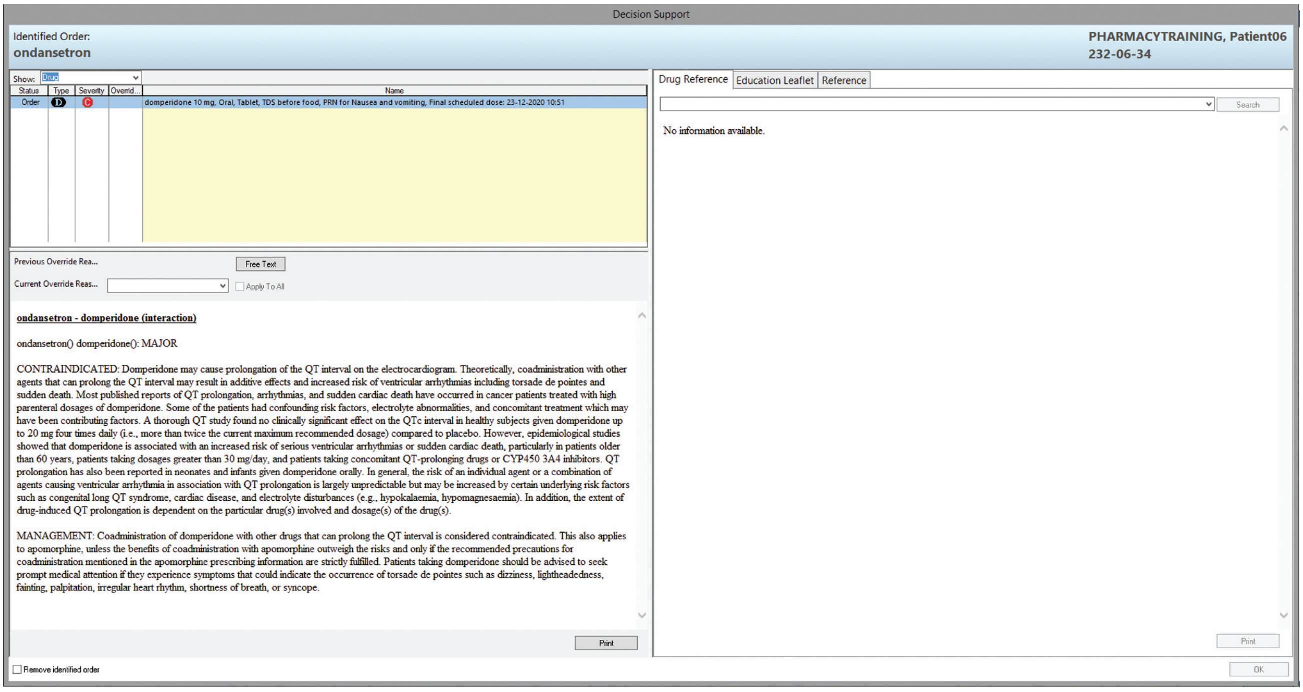

 © 2024. The Author(s).

11a. What are your thoughts about the clinical relevance of this drug–drug interaction alert?
11b. What would you do if you saw this drug–drug interaction alert for one of your patients?
11c. Does anything frustrate you about this drug–drug interaction alert?
11d. How would you improve this drug–drug interaction alert?

Scenario 10: <show alert for droperidol AND tacrolimus (QTc and TdP)>

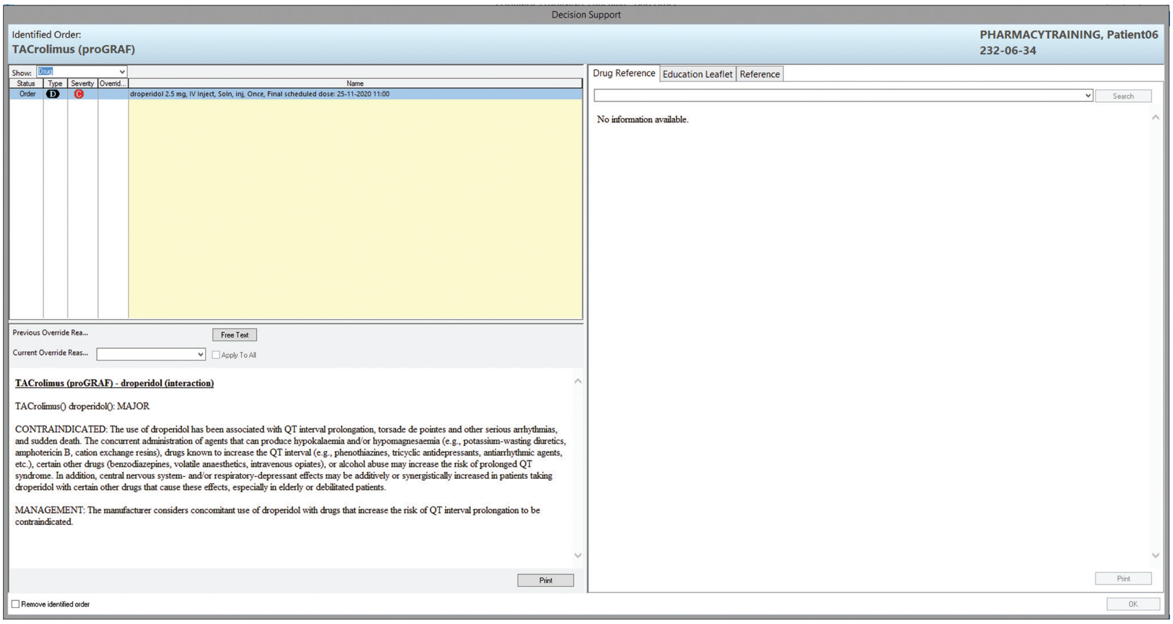

Fig 10

12a. What are your thoughts about the clinical relevance of this drug–drug interaction alert?
12b. What would you do if you saw this drug–drug interaction alert for one of your patients?
12c. Does anything frustrate you about this drug–drug interaction alert?
12d. How would you improve this drug–drug interaction alert?

Scenario 11: <show alert for droperidol AND escitalopram (QTc and TdP, EPS or NMS, SS, Psychomotor impairment)>

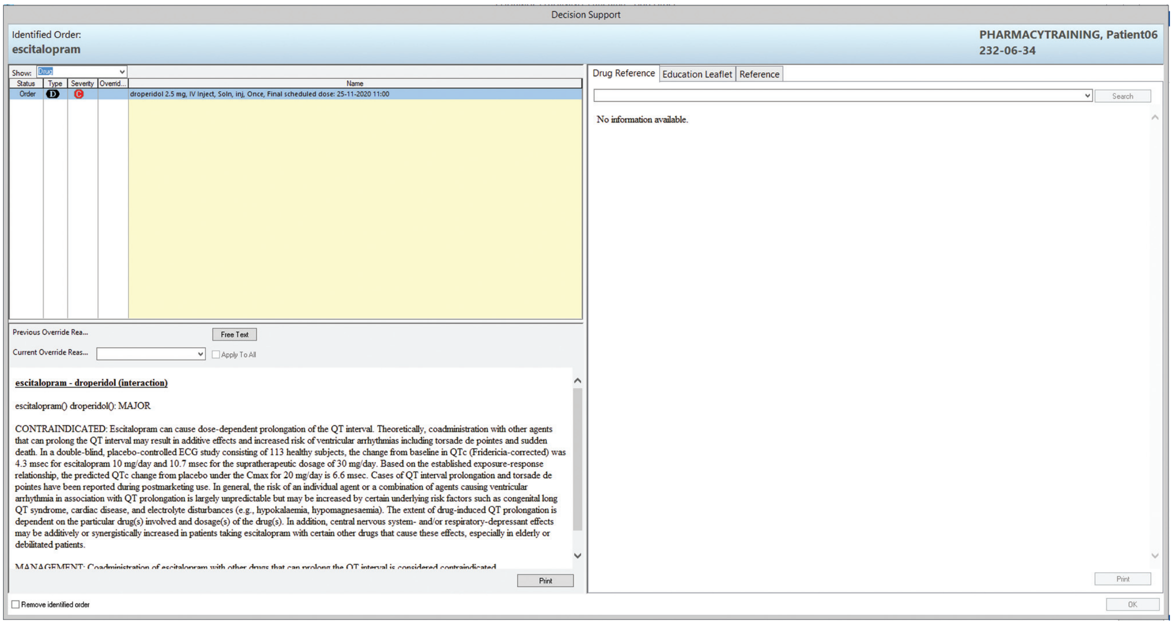

Fig 11

 © 2024. The Author(s).

13a. What are your thoughts about the clinical relevance of this drug–drug interaction alert?
13b. What would you do if you saw this drug–drug interaction alert for one of your patients?
13c. Does anything frustrate you about this drug–drug interaction alert?
13d. How would you improve this drug–drug interaction alert?

Scenario 12: <show alert for haloperidol AND quetiapine (QTc and TdP)>

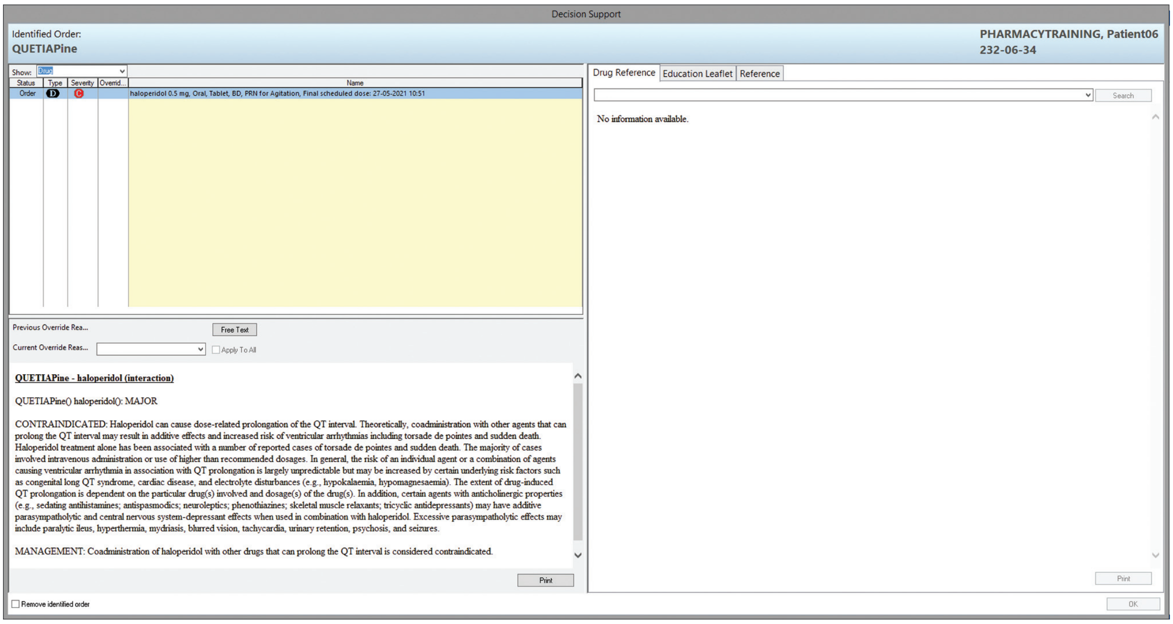

Fig 12

14a. What are your thoughts about the clinical relevance of this drug–drug interaction alert?
14b. What would you do if you saw this drug–drug interaction alert for one of your patients?
14c. Does anything frustrate you about this drug–drug interaction alert?
14d. How would you improve this drug–drug interaction alert?

Scenario 13: <show alert for escitalopram AND prochlorperazine (QTc and TdP, EPS or NMS, SS, Psychomotor impairment)>

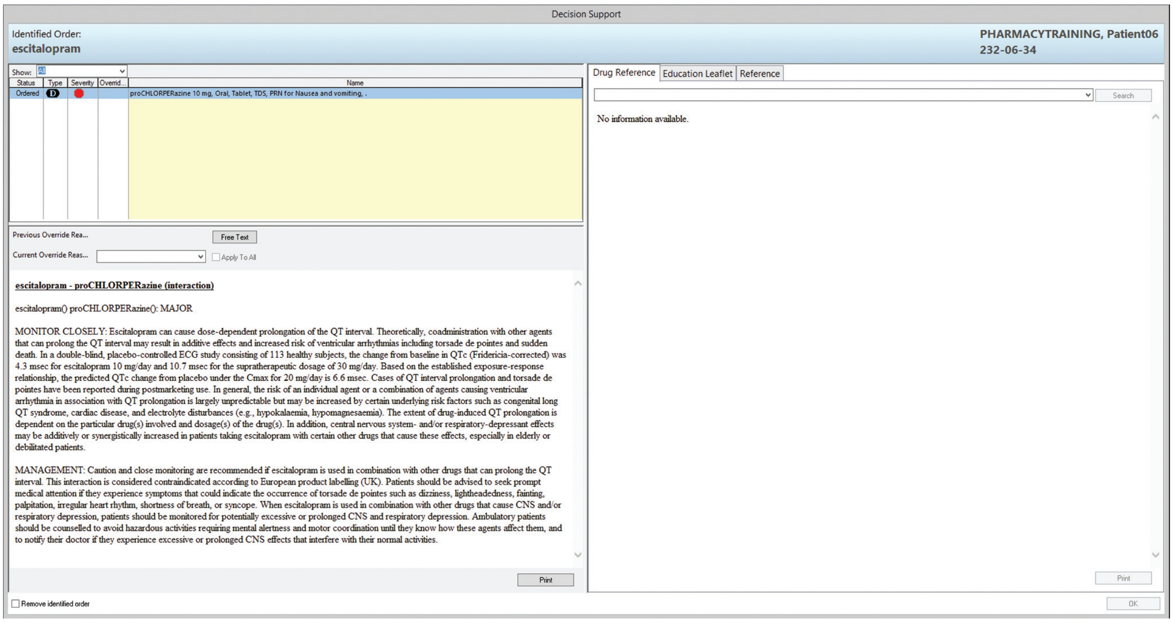

Fig 13

 © 2024. The Author(s).

15a. What are your thoughts about the clinical relevance of this drug–drug interaction alert?
15b. What would you do if you saw this drug–drug interaction alert for one of your patients?
15c. Does anything frustrate you about this drug–drug interaction alert?
15d. How would you improve this drug–drug interaction alert?

Scenario 14: <show alert for ziprasidone AND zuclopenthixol (QTc and TdP, CNS Depression)>

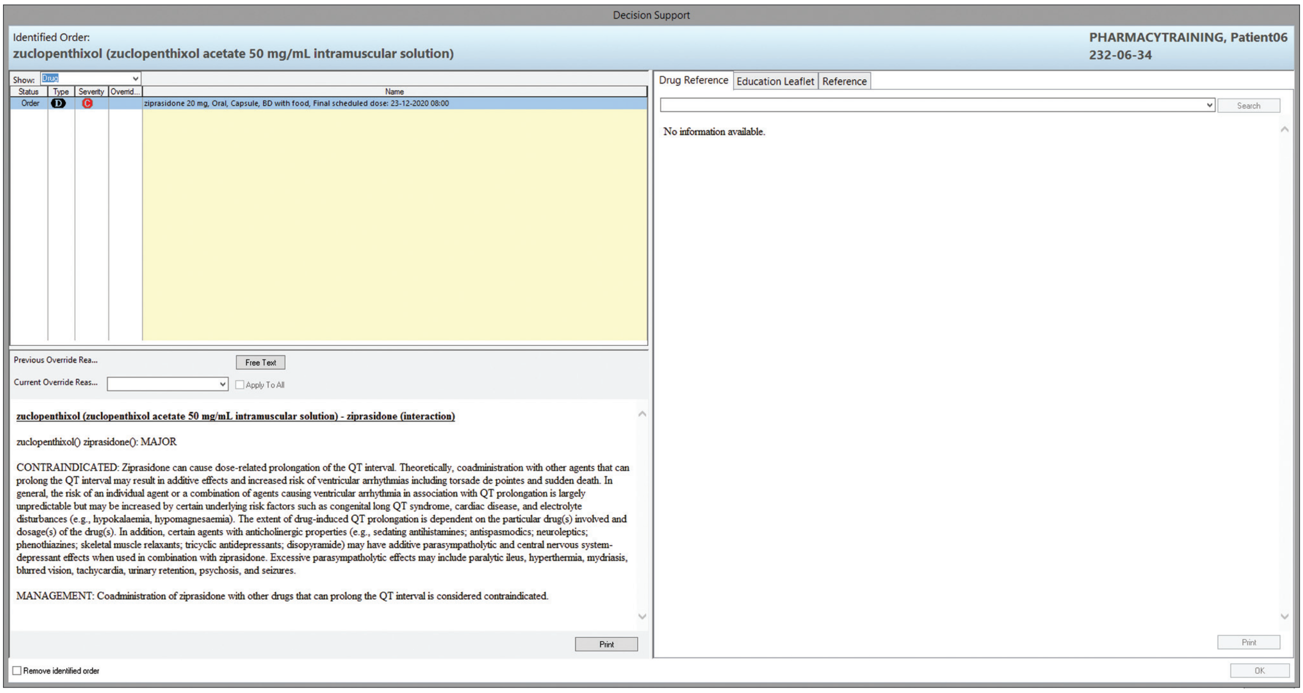

Fig 14

16a. What are your thoughts about the clinical relevance of this drug–drug interaction alert?
16b. What would you do if you saw this drug–drug interaction alert for one of your patients?
16c. Does anything frustrate you about this drug–drug interaction alert?
16d. How would you improve this drug–drug interaction alert?

 © 2024. The Author(s).

Scenario 15: <show alert for escitalopram AND quetiapine (QTc and TdP, EPS or NMS, SS, Psychomotor impairment)>

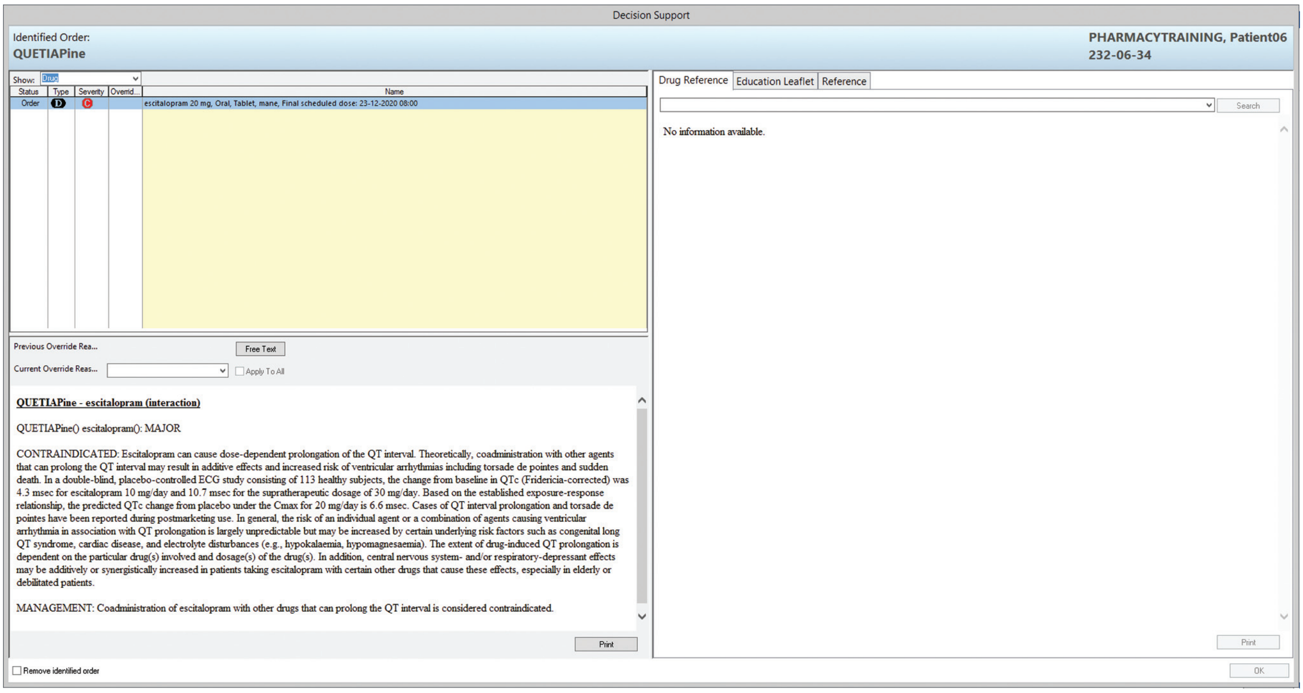

Fig 15

17a. What are your thoughts about the clinical relevance of this drug–drug interaction alert?
17b. What would you do if you saw this drug–drug interaction alert for one of your patients?
17c. Does anything frustrate you about this drug–drug interaction alert?
17d. How would you improve this drug–drug interaction alert?

Scenario 16: <show alert for buprenorphine AND haloperidol (QTc and TdP, CNS Depression)>

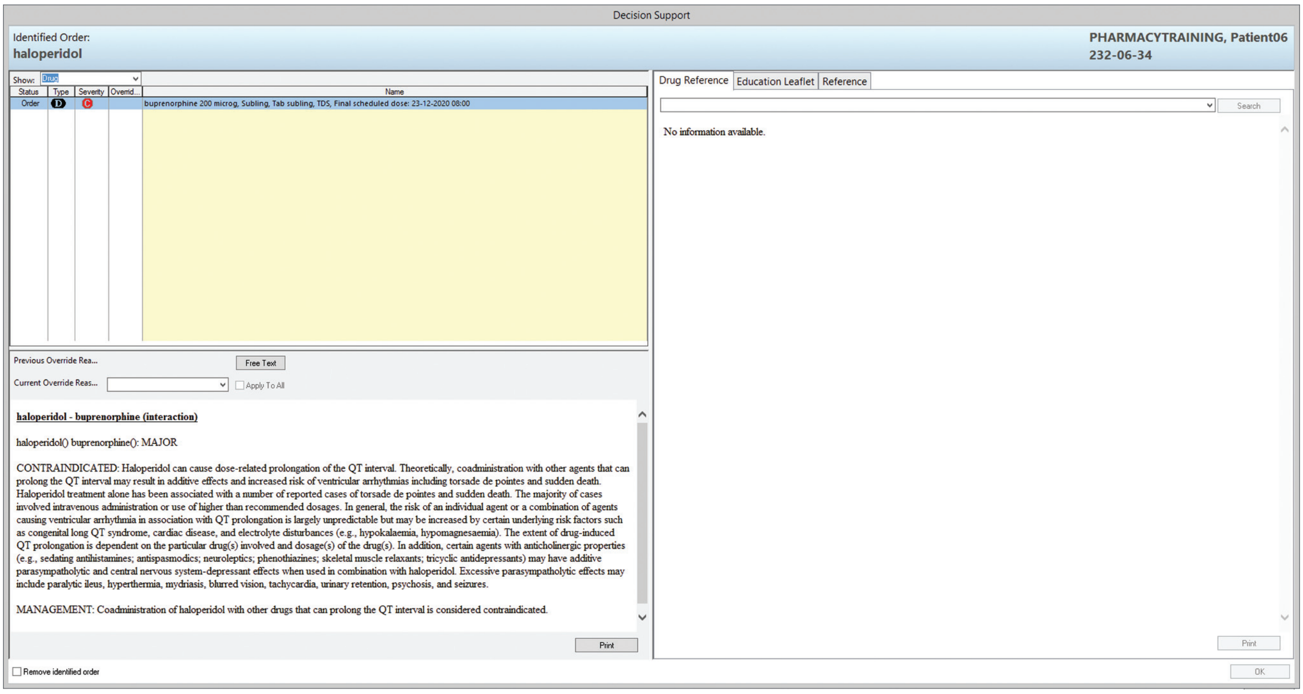

Fig 16

 © 2024. The Author(s).

18a. What are your thoughts about the clinical relevance of this drug–drug interaction alert?
18b. What would you do if you saw this drug–drug interaction alert for one of your patients?
18c. Does anything frustrate you about this drug–drug interaction alert?
18d. How would you improve this drug–drug interaction alert?

Scenario 17: <show alert for citalopram AND prochlorperazine (QTc and TdP, EPS or NMS, SS, Psychomotor impairment)>

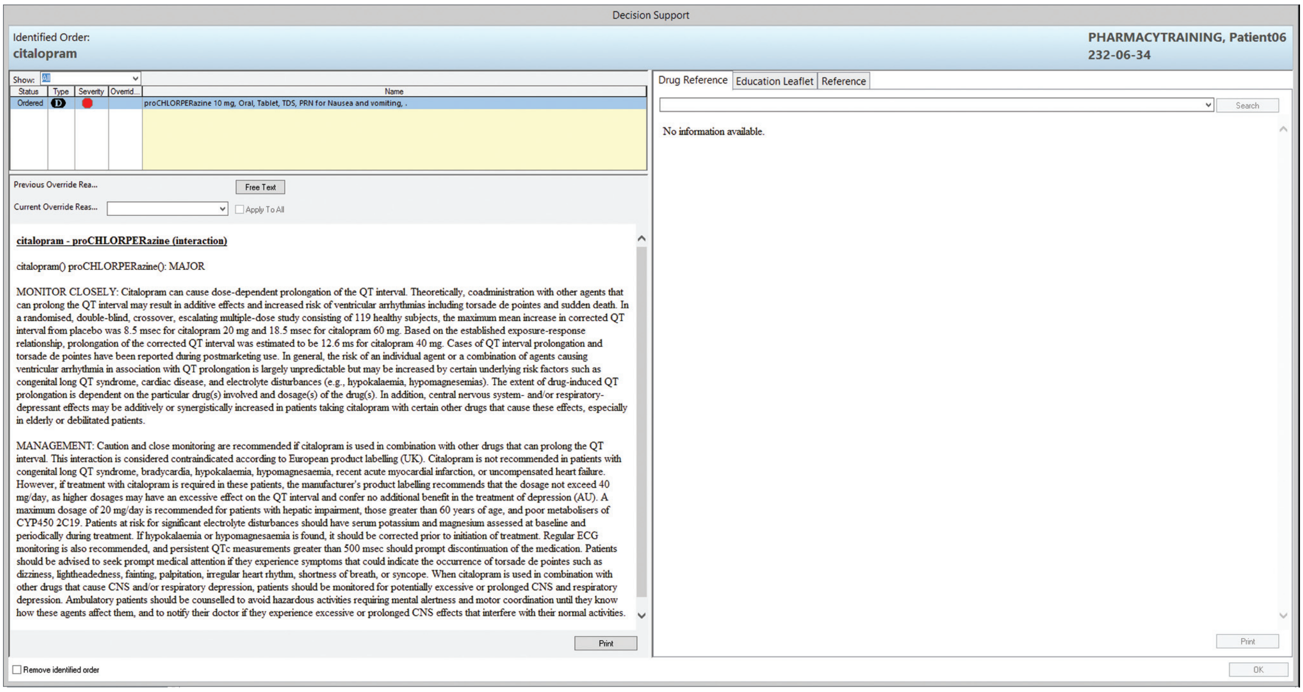

Fig 17

19a. What are your thoughts about the clinical relevance of this drug–drug interaction alert?
19b. What would you do if you saw this drug–drug interaction alert for one of your patients?
19c. Does anything frustrate you about this drug–drug interaction alert?
19d. How would you improve this drug–drug interaction alert?

 © 2024. The Author(s).

Scenario 18: <show alert for droperidol AND quetiapine (QTc and TdP)>

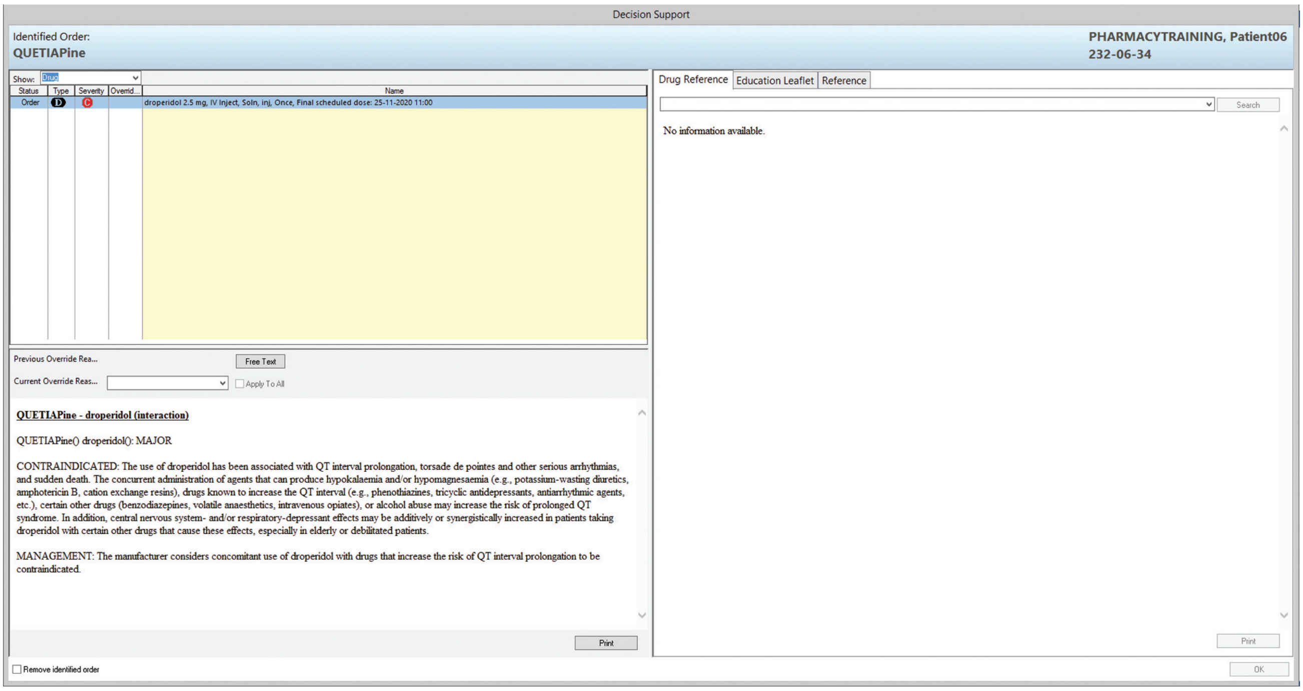

Fig 18

20a. What are your thoughts about the clinical relevance of this drug–drug interaction alert?
20b. What would you do if you saw this drug–drug interaction alert for one of your patients?
20c. Does anything frustrate you about this drug–drug interaction alert?
20d. How would you improve this drug–drug interaction alert?

Scenario 19: <show alert for droperidol AND mirtazapine (QTc and TdP, CNS Depression)>

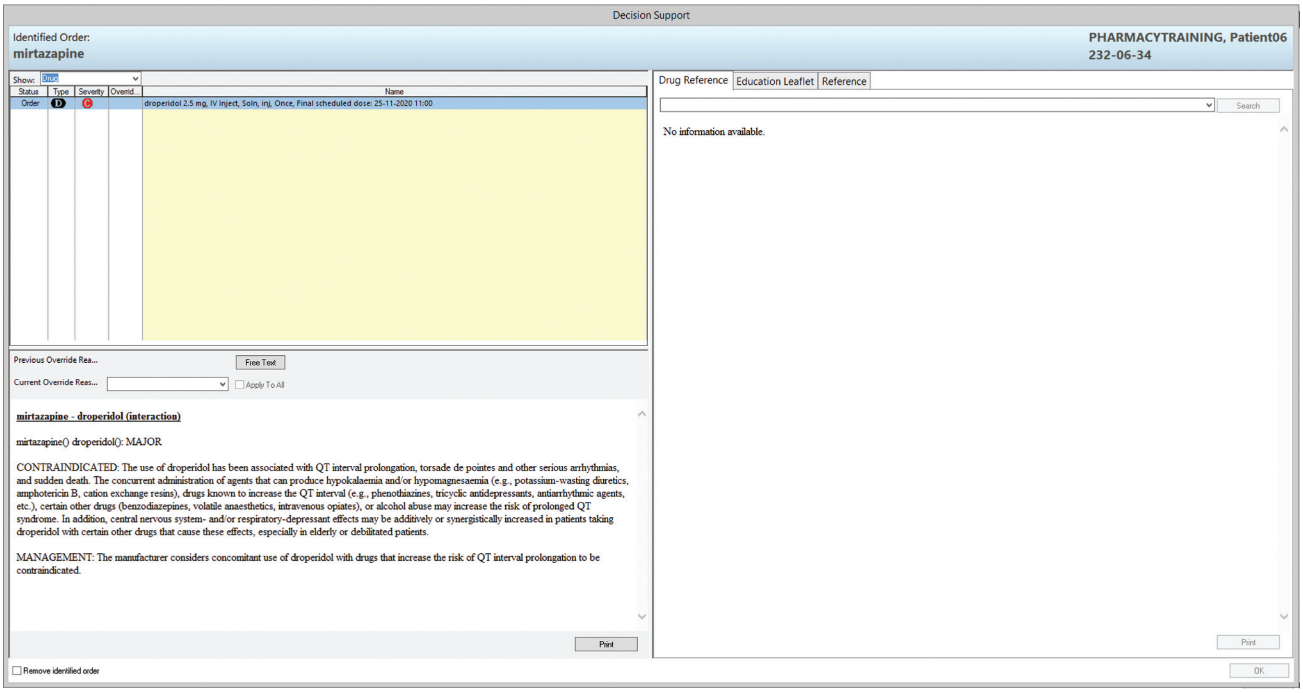

Fig 19

 © 2024. The Author(s).

21a. What are your thoughts about the clinical relevance of this drug–drug interaction alert?
21b. What would you do if you saw this drug–drug interaction alert for one of your patients?
21c. Does anything frustrate you about this drug–drug interaction alert?
21d. How would you improve this drug–drug interaction alert?

Scenario 20: <show alert for domperidone AND salbutamol (QTc and TdP)>

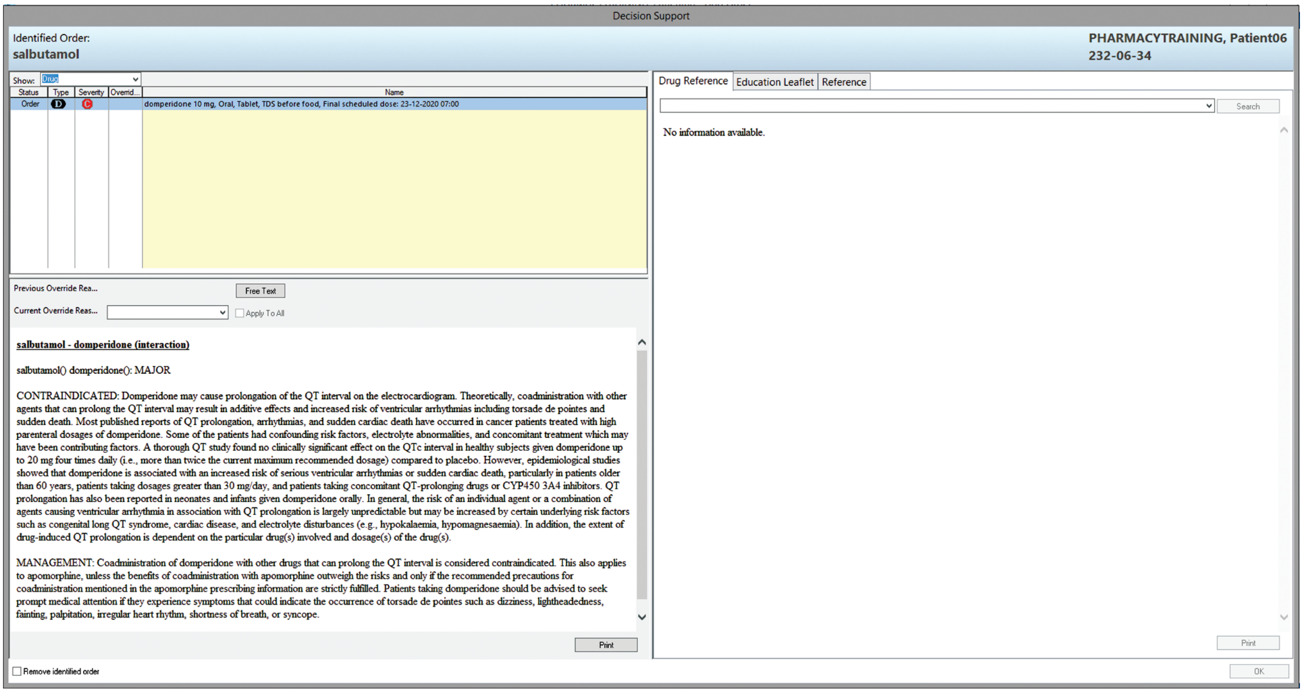

Fig 20

22a. What are your thoughts about the clinical relevance of this drug–drug interaction alert?
22b. What would you do if you saw this drug–drug interaction alert for one of your patients?
22c. Does anything frustrate you about this drug–drug interaction alert?
22d. How would you improve this drug–drug interaction alert?

III. CLOSE

23. That is the end of the interview. Do you have any concluding thoughts?

 © 2024. The Author(s).
